# Limited evidence for the effect of red color on cognitive performance: A meta-analysis

**DOI:** 10.3758/s13423-020-01772-1

**Published:** 2020-07-07

**Authors:** Timo Gnambs

**Affiliations:** 1 Leibniz Institute for Educational Trajectories, Wilhelmsplatz 3, 96047 Bamberg, Germany; 2 Johannes Kepler University Linz, Linz, Austria

**Keywords:** Red color, Cognitive performance, Intelligence, Meta-analysis

## Abstract

Red color supposedly affects cognitive functioning in achievement situations and impairs test performance. Although this has been shown for different cognitive domains in different populations and cultural contexts, recent studies including close replications failed to corroborate this effect. Reported here is a random-effects meta-analysis of 67 effect sizes (38 samples) that compared test performance after viewing red or a control color. For anagram tests and knowledge tests no significant difference between color conditions was found (Cohen’s *d* of -0.06 and -0.04); for reasoning tests the pooled effect of *d* = -0.34, 95% CI [-0.61, -0.06] indicated significantly lower scores in the red condition. The cumulative meta-analysis revealed substantially larger effects in initial studies as compared to subsequent research. After correcting for publication bias no evidential value for an effect of red color on intellectual performance was available. The review casts doubt on the existence of a robust color-priming effect in achievement situations.

**Electronic supplementary material:**

The online version of this article (10.3758/s13423-020-01772-1) contains supplementary material, which is available to authorized users.

## Introduction

Perceiving color stimuli can influence psychological functioning including cognitive performance. In a series of experiments, Elliot and colleagues (Elliot, Maier, Moller, Friedman, & Meinhardt, 2007) showed that in an achievement context presenting a small stimulus in red color as compared to another color (e.g., green or gray) significantly reduced subsequent performance on anagram tests and measures of reasoning abilities. The respective effects of red color were quite substantial. Effect sizes (Cohen’s *d*) fell between -0.51 and -1.14 (Elliot et al., 2007), which seem rather impressive given the subtle color manipulations. In one experiment Elliot et al. (2007) manipulated a small person number (sized 1.3 × 1.9 cm) that was placed on the upper right corner of each page of the test booklet by using red, green, or black ink. In another experiment, the authors briefly presented a colored rectangle (sized 12.7 × 18 cm) on the cover page before the actual test. In all experiments, viewing red color consistently led to poorer test performance as compared to exposure to another color. Although different theoretical explanations have been put forward for this phenomenon (see Elliot & Maier, 2014, for a review), available empirical evidence suggests that in an achievement context perceiving red color, due to its implicit association with caution and danger, influences achievement motivation; seeing red implicitly activates thoughts about failure and instigates avoidance motivation which, in turn, leads to poorer test performance (Maier, Elliot, & Lichtenfeld, 2008).

Following this initial research, several follow-up studies corroborated and extended these findings. For example, the negative effect of red color on cognitive performance was generalized from fluid measures of intelligence to indicators of crystallized intelligence (Gnambs, Appel, & Batinic, 2010; Gnambs, Appel, & Kaspar, 2015a). It emerged among children (Brooker & Franklin, 2016) and was also replicated in different cultural contexts (Shi, Zhang, & Jiang, 2015). These results suggest a universal effect of red color on intellectual performance that generalizes across different populations and cognitive domains. If these color effects can be substantiated, the coupling of color processing and higher cognitive performance might have important implications for the current understanding of the structure and neurological functioning of cognitive abilities. For practitioners, it might also require adapting psychological and educational assessments when constructing and administering achievement tests. To prevent memory effects or cheating, it is not uncommon to administer parallel versions of a test to examinees. If these tests are printed on differently colored paper or include items with different colors the different test versions might involuntarily bias, for example, certification programs or selection procedures, particularly if colors are not matched across different test versions.

Despite the seemingly overwhelming evidence of the negative consequences of the color red, some researchers have questioned a substantial color-performance link. For example, Larsson and von Stumm (2015) administered various cognitive measures (including reasoning and general knowledge tests) to a large sample of British adults but found no evidence for an effect of red color on test performance. Current context theories of color (Elliot & Maier, 2014) do not explain why an effect of red color should be absent among adults from the general population, although it was clearly observed among adolescents (Elliot et al., 2007). Similarly, no substantial color effect on exam performance was identified among young adults in higher education (Arthur, Cho, & Muñoz, 2016). Moreover, some authors observed effects only for male participants, whereas women seemed to be unaffected by red (e.g., Gnambs et al., 2010). Again, current theoretical models would not expect a sex-dependent impact of red; rather, color effects are assumed to be universal. More importantly, several close replications of the experiments reported in Elliot et al. (2007) were unable to reproduce the original findings (e.g., Steele et al., 2018) and related effects (e.g., cognitive effects of processing the word red; Gnambs, Kovacs, & Stiglbauer, 2020). The conflicting findings on red color effects in achievement situations are also evident in Table 1, which summarizes the initial and most recent study results for different cognitive measures. These results demonstrate that the available evidence for a detrimental effect of red color in achievement contexts is not as unequivocal as initial research might have indicated.

**Table 1:** Effects of red color on cognitive performance in early (2007–2010) and most recent studies (2015–2018)

Study	Cognitive test	Colors	*N*	Cohen’s *d*	*SE*_*d*_	*p*_*d*_
*Early studies*						
Elliot et al. (2007)						
Experiment 1	Anagrams	3	71	-0.14	0.27	.60
Experiment 2	Verbal reasoning	3	46	-1.11	0.35	< .001
Experiment 3	Verbal reasoning	3	30	-1.14	0.43	.01
Experiment 4	Numeric reasoning	3	57	-0.51	0.29	.08
Maier et al. (2008)						
Experiment 1	Numeric reasoning	2	20	-1.37	0.50	.01
Experiment 3	Numeric reasoning	2	22	-0.96	0.45	.03
Gnambs et al. (2010)^a^						
Experiment 1	General knowledge	2	40	-0.93	0.33	.01
Experiment 2	General knowledge	2	64	-0.67	0.26	.01
*Recent studies*						
Larsson & von Stumm (2015)	Verbal reasoning	2	187	-0.04^b^	0.12	.72
	General knowledge	2	187	-0.01^b^	0.13	.95
Arthur et al. (2016)						
Experiment 1	Knowledge test	2	76	0.43	0.23	.06
Experiment 2	Knowledge test	2	164	0.02	0.16	.89
Experiment 3	Knowledge test	2	87	0.08	0.21	.69
Steel et al. (2018)	Anagrams	3	421	-0.04	0.10	.68

*Note*. The earliest and most recent studies for each cognitive test are presented (all effect sizes are available in Supplementary Online Material, Section B)

*Colors* number of color conditions, *N* total sample size, *d* average effect size across different color conditions with negative effects indicating worse performance for red as compared to a control color, *SE*_*d*_ standard error for *d, p*_*d*_ *p*-value for *d*

^a^Male subsample

^b^Averaged across multiple measures

## The Present Study

In light of the conflicting empirical evidence, a meta-analysis is presented that summarizes experimental findings on the effect of red color on cognitive test performance. The review focuses on measures of anagram and reasoning test performance because they have initially been shown to be affected by red (Elliot et al., 2007) and have been used in multiple replications. Moreover, I also examined whether color effects generalize to indicators of crystalized intelligence in the form of knowledge test performance (see Gnambs et al., 2010). A particular emphasis is placed on the robustness of the available findings. Because many psychological fields seem to be plagued by false positives and failures to replicate seemingly well-known effects (Klein et al., 2018), this review seeks to establish whether the currently available research literature provides evidential value for color effects or, rather, that the reported findings are disproportionally distorted by a publication bias that makes drawing substantive conclusions infeasible.

## Method

### Meta-analytic database

Primary studies were identified on 19 August 2019 in Google Scholar using the search string *(“red color” OR “color red”) AND (“intellectual performance” OR “intellectual abilities” OR “cognitive performance” OR “cognitive abilities” OR “intelligence test”)*. Additional studies were located in four open data repositories (Open Science Framework, Harvard Dataverse, Journal of Open Psychology Data, PsychFileDrawer). Finally, studies were also retrieved by inspecting all studies referencing Elliot et al. (2007) and the references of the previously identified articles. Further details on the search process are available in Supplementary Online Material, Section A. Studies that met the following criteria were included in the meta-analysis: (a) The study was published in 2007 (the publication year of the seminal work by Elliot et al., 2007) or later. (b) The study implemented an experimental manipulation of color that (c) included red color and at least one control color (blue, green, black, gray, or white). (d) Respondents were randomly assigned to the experimental conditions. (e) Cognitive performance was measured using one of the following tests: (i) anagram tests that required respondents to reorder a set of scrambled letters into a meaningful word, (ii) reasoning tests that required respondents to apply a logical rule deduced from a stimulus set to identify a correct response (e.g., word analogies) or generate a new response (e.g., number sequences), or (iii) knowledge tests that required respondents to retrieve stored information from their long-term memory (e.g., general knowledge tests). Cognitive measures that could not be clearly assigned to one of these categories (e.g., reading comprehension) were not considered. (f) The cognitive test was administered after the color manipulation. (g) Additionally, relevant effect sizes or statistics to compute an effect size and (h) the sample size must have been reported. Studies were excluded if they (a) reported on clinical samples with diagnosed psychiatric symptoms, (b) reported exclusively on subjective performance assessments (instead of objective test scores), (c) used color words as experimental manipulation instead of presenting actual color stimuli, or (d) used a mixed color condition (e.g., pink instead of red). After applying these criteria, 22 publications reporting on 38 independent samples were available. The characteristics of these samples including the coded effect sizes are summarized in Supplementary Online Material, Section B. Studies that were excluded from the meta-analysis (including the reason for their exclusion) are given in Supplementary Online Material, Section A.

### Meta-analytic procedure

The effect size was the standardized mean difference between red color and a control color. The effect sizes were coded in such a way that negative values indicated lower test scores in the red condition. The exact formulas for the calculation of the effect sizes and their sampling variances are given in Supplementary Online Material, Section C. The effect sizes were pooled using a random-effects model with a restricted maximum likelihood estimator using the *metafor* software version 2.1-0 (Viechtbauer, 2010). To account for sampling error, each effect size was weighted by the inverse of its variance. Because some studies provided more than one effect size obtained for different control colors or different cognitive measures, a multivariate meta-analysis was specified that acknowledged the sampling covariances between the effect sizes (Gleser & Olkin, 2009). The homogeneity of the effects sizes was tested using the χ^2^-distributed *Q*-statistic and quantified using *I*^2^, which indicates the percentage of the total variance in observed effects due to random variance. Moderators were evaluated using meta-regression analyses that calculated the χ^2^-distributed omnibus test statistic *Q*_*m*_ and the percentage reduction in random variance (*R*^2^).

Analyses of publication bias were based on the within-sample averaged effect sizes (the respective formulas are given in Supplementary Online Material, Section C) and conducted in seven ways: Funnel plot asymmetry was studied with Begg and Mazumdar’s (1994) rank correlation test, PET / PEESE analyses (Stanley, 2017), and the regression test by Peters, Sutton, Jones, and Rushton (2006). Following Pustejovsky and Rodgers (2019), the latter two were also modified by substituting the sampling variances of the effect sizes with a function of the respective sample sizes. Finally, two methods were selected that do not hinge on funnel plot asymmetry: selection models (Vevea & Woods, 2005) and puniform* analyses (vvan Aert & van Assen, 2020). The goal of these analyses was to determine whether the published findings provide evidence for a true phenomenon or, rather, are no more than a reflection of publication bias.

## Results

The meta-analysis included 22 studies that reported on 38 independent samples and provided 67 effect sizes. Each sample contributed between 1 and 5 (*Mdn* = 2) effects that contrasted red color with a control color. The effect sizes were based on a median of 76 respondents (*Min* = 16, *Max* = 282). Most samples were drawn in the USA (45%), Germany (24%), or England (16%). The median proportion of female participants was 69% and the mean age of the samples ranged from 17 to 35 years (*Mdn* = 22). About 59% of studies were published in peer-reviewed journals, whereas the rest represented unpublished work in the form of theses, conference presentations, or research reports.

### Pooled effects for red color

**Fig. 1:**
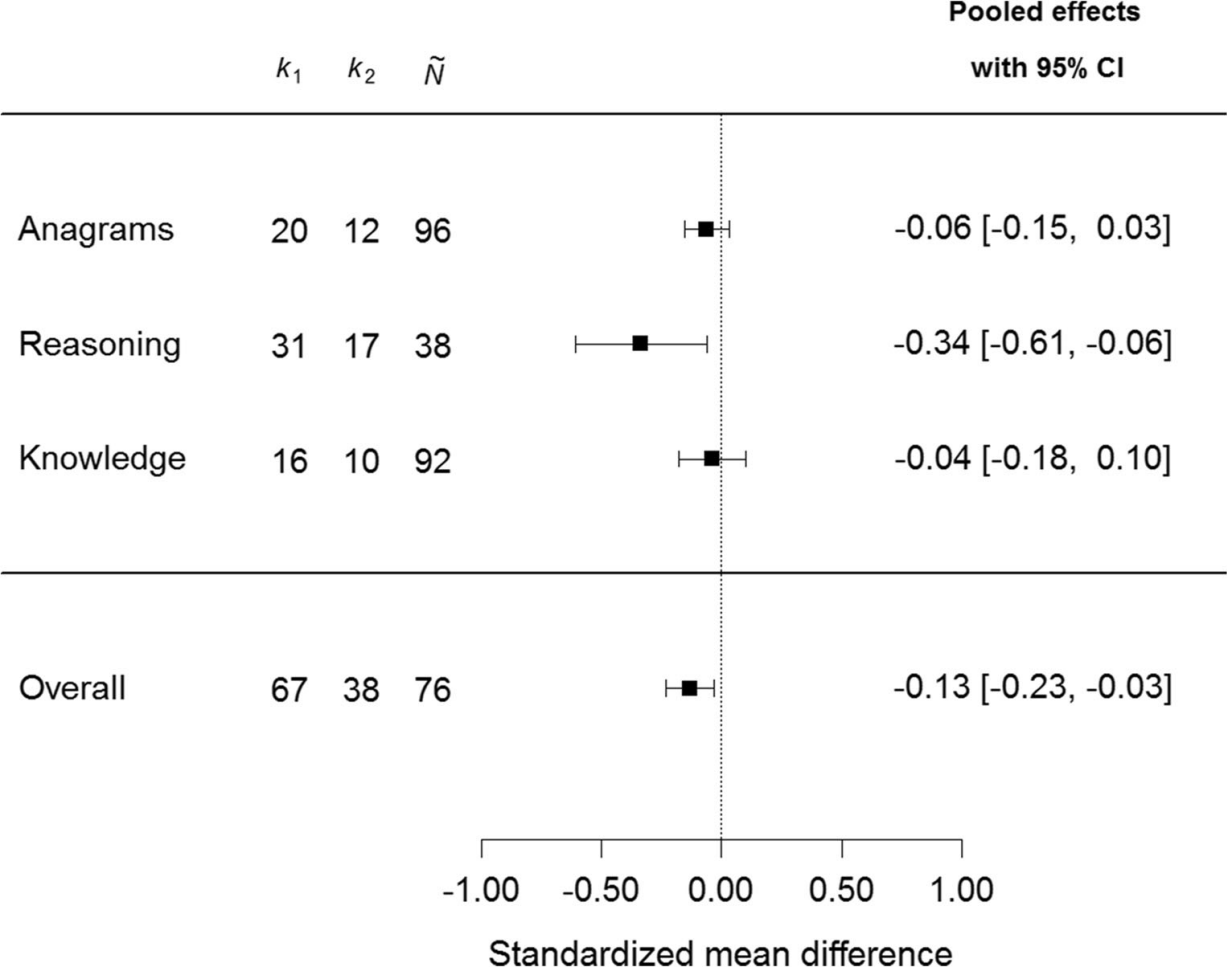
Forest plot for red color effects by cognitive measure. Negative effects indicate worse performance for red color. *k*_1_ number of effect sizes, *k*_2_ number of samples, $\overset{\sim }{N}$ median sample size per effect. Detailed meta-analytic results are available in Supplementary Online Material, Section D

The uncorrected standardized mean difference between red and a control color was *M*(*d*) = -0.16 (*SD* = 0.47) and, thus, indicated a rather small effect. Even after acknowledging sampling error the pooled effect of Δ = -0.13, 95% CI [-0.23, -0.03] revealed only a small, albeit significant (*p* = .011), difference between colors. However, there was moderate variability between samples, τ^2^ = 0.04, *p* < .001, *I*^2^ = .49. Therefore, several moderators were examined. The choice of control color (green, blue, gray, other) had no significant effect and did not explain the observed heterogeneity, *Q*_*m*_(*df* = 3) = 1.05, *p* = .789, *R*^2^ < .001. Similarly, whether the two colors were matched on hue and lightness, the color manipulation was presented on paper or a computer screen, or the color manipulation was presented only before (i.e., on the cover page) or also during the assessment (i.e., on each page) exhibited no moderating effects, *Q*_*m*_(*df* = 3) = 2.05, *p* = .561, *R*^2^ < .001. In contrast, descriptive comparisons for the cognitive measures (anagrams, reasoning, knowledge) showed impaired performance for red color on reasoning tests, Δ = -0.34, *p* = .016, 95% CI [-0.61, -0.06], whereas for anagram tests, Δ = -0.06, *p* = .184, 95% CI [-0.15, 0.03], and knowledge tests, Δ = -0.04, *p* = .598, 95% CI [-0.18, 0.10], no reduction in test performance under the influence of red was observed. However, the omnibus test did not corroborate the observed differences statistically, *Q*_*m*_(*df* = 2) = 2.22, *p* = .329, *R*^2^ < .001. Figure 1 summarizes the pooled effects for the different cognitive measures, and detailed numeric results for the meta-analytic effects in different subgroups (including sensitivity analyses) are available in Supplementary Online Material, Section D.

### Replication of red color effects

**Fig. 2:**
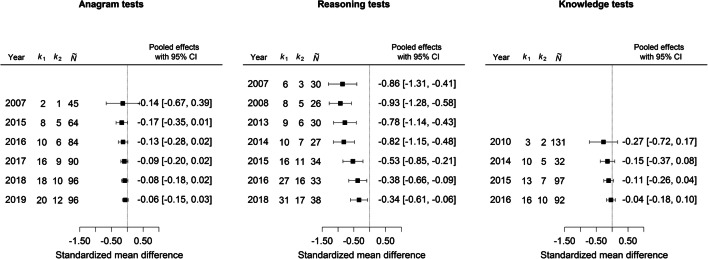
Cumulative meta-analyses of red color effects by year. Negative effects indicate worse performance for red color. *k*_1_ number of effect sizes, *k*_2_ number of samples, $\overset{\sim }{N}$ median sample size per effect. Detailed meta-analytic results are available in Supplementary Online Material, Section D

To examine whether the initially observed effects (Elliot et al., 2007; Maier et al., 2008) could be reproduced in later studies, cumulative meta-analyses (Clarke et al., 2014) for each cognitive measure sequentially pooled subsets of studies, starting with all findings published in 2007 and adding the remaining studies step-by-step according to their publication year. The chronological pooling of results can provide information about the consistency of the results and whether the available experiments allow for robust conclusions regarding the color effects. The results of the cumulative meta-analyses for the anagram, reasoning, and knowledge tests in Fig. 2 show a general decline of the pooled effects over time. For anagram and knowledge tests, at no time point was a significant effect of red color observed (left and right plots). In contrast, for reasoning tests the pooled effects at each time point were significant, but gradually became smaller over time (middle plot). Whereas initial studies found substantial color effects for reasoning tests (Δ = -0.86, *p* < .001, 95% CI [-1.31, -0.41]), subsequent findings identified a more modest pooled effect of Δ = -0.34, *p* = .016, 95% CI [-0.61, -0.06]. Thus, support for red’s impact on cognitive performance was limited to initial research, while follow-up studies tended to provide far less evidence.

### Publication bias

**Fig. 3:**
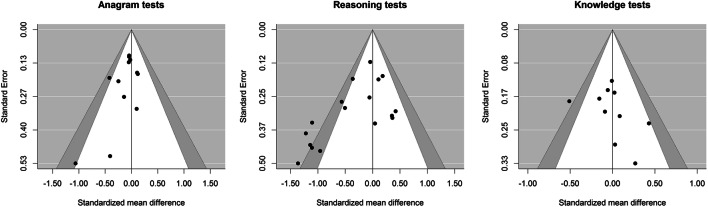
Funnel plots with 95% (white) and 99% (gray) confidence intervals for anagram, reasoning, and knowledge tests

The presence and consequence of a potential publication bias were examined in several ways (see Supplementary Online Material, Section E for details). The funnel plots in Fig. 3 indicated asymmetric shapes for anagram and reasoning tests; non-significant, small-study effects seemed to be systematically missing. The results of the different publication bias analyses are summarized in Table 2. The rank correlation test, *r* = -.24 (*p* = .036) indicated a significant (*p* < .05) funnel plot asymmetry when examining all effect sizes for the three cognitive measures. Similarly, the three regression tests also suggested skewed funnel plots (see Table 2). More importantly, for all three regression models the pooled effects adjusted for publication bias were not significantly different from zero and estimated effects close to Δ = 0.00. Moreover, the selection models indicated significant publication bias and yielded slightly positive adjusted effects (about Δ = 0.03). Finally, an examination of the *p*-value distributions of the observed effect sizes (van Aert & van Assen, 2020) found no evidential value for an effect of red color on cognitive performance (about Δ = -0.02). Because reasoning tests were the only cognitive measures exhibiting a somewhat larger pooled effect as compared to the other cognitive measures (see above), all tests for publication bias were repeated in this subgroup (see Table 2). All tests indicated significant (*p* < .05) publication bias. Depending on the statistical method, the pooled effects adjusted for publication bias were close to zero (selection models, puniform*) or even slightly positive (regression models), suggesting an effect in the opposite direction. Taken together, the different analyses suggest that publication bias seemed to have distorted the publicly available research findings on red color and cognitive performance. After correcting for publication bias, most analyses showed no evidence of color effects.

**Table 2:** Summary of tests for publication bias

		Adjusted effect	Test for publication bias
*All cognitive measures*: *k* = 38, Δ_uc_ = -0.13 (*p* = 0.011)			
1.	Rank correlation^a^		*r* = -.24 (*p* = .036)
2.	Peter et al.’s (2006) regression test	Δ = 0.03 (*p* = .604)	*B* = -13.43 (*p* = .009)
3.	PET/PEESE^b^	Δ = 0.04 (*p* = .513)	*B* = -3.29 (*p* = .002)
4.	Modified PET/ PEESE^c^	Δ = 0.03 (*p* = .604)	*B* = -3.36 (*p* = .009)
5.	Selection models^d^	Δ = 0.03 (*p* = .419)	χ^2^ = 29.53 (*p* < .001)
6.	Modified selection models^c^	Δ = 0.01 (*p* = .702)	χ^2^ = 26.48 (*p* < .001)
7.	puniform*^e^	Δ = -0.02 (*p* = .678)	*L*_*pb*_ = 15.49 (*p* < .001)
*Anagram tests*: *k* = 12, Δ_uc_ = -0.06 (*p* = 0.184)			
1.	Rank correlation^a^		*r* = -.27 (*p* = .250)
2.	Peter et al.’s (2006) regression test	Δ = -0.01 (*p* = .900)	*B* = -9.28 (*p* = .095)
3.	PET/PEESE^b^	Δ = 0.00 (*p* = .983)	*B* = -2.31 (*p* = .069)
4.	Modified PET/PEESE^c^	Δ = -0.01 (*p* = .900)	*B* = -2.32 (*p* = .095)
5.	Selection models^d^	Δ = -0.01 (*p* = .785)	χ^2^ = 2.76 (*p* = .331)
6.	Modified selection models^c^	Δ = -0.02 (*p* = .678)	χ^2^ = 2.49 (*p* = .114)
7.	puniform*^e^	Δ = -0.04 (*p* = .422)	*L*_*pb*_ = 0.13 (*p* = .936)
*Reasoning tests*: *k* = 17, Δ_uc_ = -0.34 (*p* = .016)			
1.	Rank correlation^a^		*r* = -.43 (*p* = .017)
2.	Peter et al.’s (2006) regression test	Δ = 0.17 (*p* = .324)	*B* = -23.44 (*p* = .021)
3.	PET/PEESE^b^	Δ = 0.14 (*p* = .333)	*B* = -5.06 (*p* = .009)
4.	Modified PET/PEESE^c^	Δ = 0.17 (*p* = .324)	*B* = -5.86 (*p* = .021)
5.	Selection models^d^	Δ = 0.08 (*p* = .175)	χ^2^ = 29.50 (*p* = .033)
6.	Modified selection models^c^	Δ = 0.05 (*p* = .444)	χ^2^ = 23.92 (*p* < .001)
7.	puniform*^e^	Δ = 0.03 (*p* = .704)	*L*_*pb*_ = 6.78 (*p* = .034)
*Knowledge tests*: *k* = 10, Δ_uc_ = -0.04 (*p* = .598)			
1.	Rank correlation^a^		*r* = .29 (*p* = .291)
2.	Peter et al.’s (2006) regression test	Δ = -0.21 (*p* = .200)	*B* = 19.80 (*p* = 252)
3.	PET/PEESE^b^	Δ = -0.17 (*p* = .242)	*B* = 3.72 (*p* = .308)
4.	Modified PET/PEESE^c^	Δ = -0.21 (*p* = .200)	*B* = 4.95 (*p* = .252)
5.	Selection models^d^	Δ = -0.02 (*p* = .813)	χ^2^ = 0.80 (*p* = .371)
6.	Modified selection models^c^	Δ = -0.02 (*p* = .806)	χ^2^ = 0.79 (*p* = .375)
7.	puniform*^e^	Δ = -0.01 (*p* = .850)	*L*_*pb*_ = 0.86 (*p* = .649)

*Note*. All analyses are based on the within-sample averaged sample sizes (see Supplementary Online Material, Section C)

Δ indicates pooled effect adjusted for publication bias, Δ_uc_ indicates unadjusted pooled effect, *k* number of independent effect sizes, *B* unstandardized regression weight, χ^2^ test statistic for publication bias with 1 degree of freedom, *L*_*pb*_ test statistic for publication bias

^a^Begg and Madzumdar (1994)

^b^Stanley (2017)

^c^Substitutes the sampling variances of *d* with a function of the sample size (Pustejovsky & Rodner, 2019)

^d^Vevea and Woods (2005)

^e^van Aert and van Assen (2020)

## Discussion

Recent years have seen an increased interest in research on color and psychological functioning (Elliot & Maier, 2014). In achievement situations, red color has been linked to avoidance motivation and reduced test performance (Maier et al., 2008). Respective effect sizes of Cohen’s *d* of 1 and above indicated that seemingly unimportant variations in inconspicuous color stimuli might have a substantial impact on cognitive functioning. The purpose of this review was to summarize empirical evidence and establish whether the available research literature provides evidential value for effects of red color on intellectual performance. For anagram tests and knowledge tests no such evidence was found. Test performance was not affected by red color. In contrast, performance on reasoning tests was significantly impaired after viewing red. However, the size of the effect (Δ = -0.34) was less than half the effect size initially reported (Elliot et al., 2007). Importantly, substantial effects were only observed in initial studies, whereas most recent research found little evidence for color effects, despite including larger samples.

Similar decline effects have been previously observed for other popular phenomena in social psychology. Most recently, a large-scale replication attempt of 28 classical and contemporary findings in psychology showed that, on average, effect sizes of direct replications were less than half the size of the original studies (Klein et al., 2018). This shows that the impact of color on achievement might have been overstated so far. This has important implications for future studies because the identification of small effects requires substantially larger samples than have been typically adopted in color psychology. The median sample size in the present review was 76; for reasoning tests typical samples included even half this number. With these samples the power to identify the pooled effect reported above was about 18%. Thus, color psychology seems to suffer from similar power problems to many other psychological fields (e.g., Lamberink et al., 2018; Szucs & Ioannidis, 2017). Because effect sizes that are reported in published studies may be substantially exaggerated when based on very small samples, the pooled effect derived in this meta-analysis may, in fact, represent an overestimation of the real effect (cf. Nuijten, van Assen, Veldkamp, & Wicherts, 2015). Indeed, bias analyses found a substantial number of negative findings missing from the available research literature. After correcting for publication bias, limited evidential value for an effect of red color on intellectual performance remained.

### Implications for red color in achievement situations

At present, there is little cause for concern for practitioners and educators that test performance might be systematically affected by minor color variations before or during a test. The failure to corroborate a robust effect of red color could indicate that the published findings supporting respective color effects represented false positives and, in fact, red color has no impact on intellectual performance. On the other hand, it is also possible that color effects exist, but they are difficult to identify (see Elliot, 2019, for a methodological critique of color research). For example, unknown changes in sample compositions between the original and the follow-up studies might have contributed to the failure to replicate the red color effect. However, recent large-scale replications including dozens of sampling contexts highlighted that for many psychological phenomena replication success showed little variation across samples (Klein et al., 2018).

Several strategies might help to move on and improve the robustness of findings on red color in achievement contexts. Most importantly, this includes emphasizing statistical power to be able to identify more modest effects. Similarly, adopting open practices and sharing experimental details would facilitate close replications of successful research studies. Generally, color research needs to adapt its research strategies and embrace the new standards that have been advocated for psychological research in recent years (e.g., Munafò et al., 2017). High-powered multi-lab collaborations (see Moshontz et al., 2018) might yet allow identifying robust color effects that generalize across samples and research teams.

### Limitations

The scope of the reported findings might be constrained by several methodological decisions during the meta-analytic review. For example, the literature search did not include an open call in mailing lists for unpublished studies. However, it is unlikely that a substantial body of unpublished research showing substantial color effects exists that might alter the conclusions of the meta-analysis. Typically, nonsignificant, small effects remain unpublished that would further corroborate the identified lack of effect. Moreover, the classification of cognitive measures into anagram, reasoning, and knowledge tests might be open for debate in some situations. For example, reading competence tests might have been considered a form of knowledge test instead of excluding these measures. Thus, a different classification scheme would have altered the body of effect sizes available for the meta-analysis. However, these cases would be rare for the present meta-analysis.

### Constraints of generality

The present meta-analysis found limited evidence for impaired intellectual performance when seeing red for measures of reasoning, anagram, and knowledge test performance. Although there is little reason to believe that red color effects might be more robust in other cognitive domains such as working memory (Elliot, Payen, Brisswalter, Cury, & Thayer, 2011) or selective attention (Lindsey et al., 2010), it would be premature to generalize the reported findings to other types of cognitive performance or even to non-cognitive outcomes (e.g., risk taking; Gnambs, Appel, & Oeberst, 2015b). For example, it has been shown that negative affective states such as anxiety can impair executive functioning (Shields, Moons, Tewell, & Yonelinas, 2016). If red color evokes negative affectivity in performance situations, red color might have a stronger impact in these domains as compared to those studied in the present meta-analysis. Furthermore, most studies included in the meta-analysis were conducted in testing situations that had no individual consequences for the respondents. It is unclear whether red color effects can also be expected when respondents are motivated enough to perform well on a test (e.g., in school exams or employment testing). Preliminary evidence (e.g., Arthur et al., 2016; see also Supplementary Online Material, Section D) indicates that red color effects (if they exist) might be limited to low-stakes, laboratory research and do not generalize to applied settings.

### Conclusion

A review of the available research findings on red color and cognitive functioning found little evidence for the assumption that red would impair test performance. After viewing red no statistically different achievement on anagram tests or knowledge tests was observed as compared to a control color. For reasoning tests an effect of medium size was observed. Substantially larger effects were found in initial studies as compared to subsequent research. After correcting for confounding publication bias no effect of red color on reasoning test performance remained. At present, the available research findings provide little evidence for a robust color-priming effect in achievement situations.

#### Open practices statement

 The data and materials for the meta-analysis are available at 10.17605/OSF.IO/NHMSC. The meta-analysis was not preregistered.

## Electronic supplementary material

ESM 1 (DOCX 295 kb)

ESM 2 (DOCX 94.7 kb)

ESM 3 (DOCX 97.1 kb)

ESM 4 (DOCX 67.8 kb)

ESM 5 (DOCX 194 kb)
