# Supplementary material for: Limited evidence for the effect of red color on cognitive performance: A meta-analysis
Source: Psychon Bull Rev. 2020 Jul 7;27(6):1374–82. doi: 10.3758/s13423-020-01772-1 (PMC7704521; doi:10.3758/s13423-020-01772-1)
Supplement: Supplementary file 1 — (DOCX 295 kb) [file 13423_2020_1772_MOESM1_ESM.docx]

Supplement A: Search and Coding Process

Content

Flow Chart of Literature Search 2

Reasons for Exclusion of Studies 3

References for Excluded Studies 4

References for Included Studies 8

Coding Process of Primary Studies 11

# Flow Chart of Literature Search


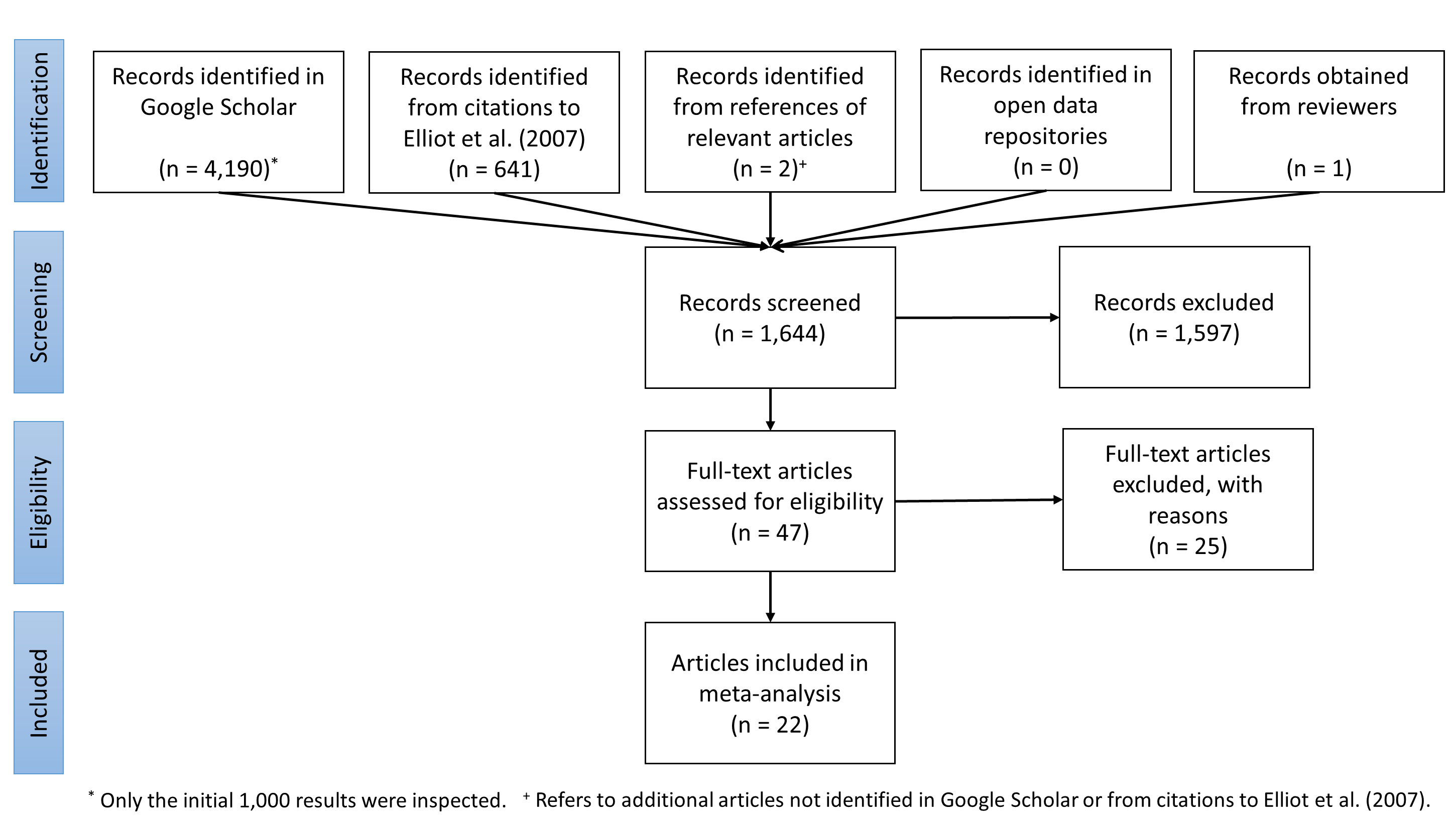


*Figure A1*. Flow chart of literature search.

# Reasons for Exclusion of Studies

The literature search identified some studies that reported on cognitive outcomes but did not meet various inclusion criteria. Therefore, the studies listed in Table A2 were excluded from the meta-analysis. Moreover, sample 3 in Smajic et al. (2014) was excluded because unreasonable large standard deviations were reported that were suspected to represent a reporting error.

Table A2.

*Studies Excluded from the Meta-Analysis*

| Study | Reason for exclusion |
| --- | --- |
| Al-Ayash et al. (2015) | No relevant cognitive measure |
| Brooker & Franklin (2016) | No relevant cognitive measure |
| Buiks (2013) | No relevant cognitive measure |
| Clary et al. (2007) | Red color not included |
| Duggan (2009) | Red color not included |
| Elliot et al. (2011) | No relevant cognitive measure |
| Fikrlova et al. (2019) | No relevant cognitive measure |
| Fordham & Hayes (2009) | Red color not included |
| Hatta et al. (2002) | Publication year before 2007 |
| Holocher (2013) | No relevant cognitive measure |
| Jung et al. (2011) | No relevant cognitive measure |
| Küller et al. (2009) | No relevant cognitive measure |
| Kumi et al. (2013) | Red color not included |
| Mehta & Zhu (2009) | No relevant cognitive measure |
| Rutchick et al. (2010) | No relevant cognitive measure |
| Lichtenfeld et al. (2012) | No relevant cognitive measure |
| Lichtenfeld et al. (2009) | No presentation of colors |
| Meyer & Bagwell (2012) | Red color not included |
| Schmidt et al. (2013) | Relevant statistics not reported |
| Steele (2014) | No relevant cognitive measure |
| Tal et al. (2008) | Relevant statistics not reported |
| Thorstenson (2012) | Software error led to biased measurements |
| Xia et al. (2016) | No relevant cognitive measure |
| Yamazaki (2010) | Relevant statistics not reported |
| Yamazaki & Eto (2011) | Relevant statistics not reported |

# References for Excluded Studies

Al‐Ayash, A., Kane, R. T., Smith, D., & Green‐Armytage, P. (2016). The influence of color on student emotion, heart rate, and performance in learning environments. *Color Research & Application, 41*, 196-205. <https://doi.org/10.1002/col.21949>

Brooker, A., & Franklin, A. (2016). The effect of colour on children's cognitive performance. *British Journal of Educational Psychology*, *86*, 241-255. <https://doi.org/10.1111/bjep.12101>

Buiks, T. J. M. (2013). *The influence of background color and type of graph on information retrieval from graphs* (Unpublished master’s thesis). Tilburg University, Netherlands.

Clary, R., Wandersee, J., & Elias, J. S. (2007). Does the color-coding of examination versions affect college science students’ test performance? Countering claims of bias. *Journal of College Science Teaching, 37*, 40-47.

Duggan, M. S. (2009). *A critical examination of the effects of colored paper on the academic achievement of fourth graders in reading comprehension and vocabulary* (Unpublished doctoral thesis). Auburn University, AL.

Elliot, A. J., Payen, V., Brisswalter, J., Cury, F., & Thayer, J. F. (2011). A subtle threat cue, heart rate variability, and cognitive performance. *Psychophysiology, 48*, 1340-1345. <https://doi.org/10.1111/j.1469-8986.2011.01216.x>

Fikrlova, J., Cechova, L., Lebedova, T., Pycha, P., Sesulkova, A., Prochazka, J., & Vaculik, M. (2019). The power of red: The influence of colour on evaluation and failure: A replication. *Acta Psychologica, 198*. <https://doi.org/10.1016/j.actpsy.2019.102873>

Fordham, D. R., & Hayes, D. C. (2009). Worth repeating: Paper color may have an effect on student performance. *Issues in Accounting Education*, *24* 187-194. <https://doi.org/10.2308/iace.2009.24.2.187>

Hatta, T., Yoshida, H., Kawakami, A., & Okamoto, M. (2002). Color of computer display frame in work performance, mood, and physiological response. *Perceptual and Motor Skills, 94*, 39-46. <https://doi.org/10.2466/pms.2002.94.1.39>

Holocher, S. (2013). *Ein Proband sieht Rot: Der Effekt von Farbreizen auf die Lernleistung* [A subject sees red: The effect of color stimuli on learning outcomes] (Unpublished bachelor’s thesis). Osnabruck University, Germany.

Jung, I., Kim, M. S., & Han, K. (2011). Red for romance, blue for memory. In C. Stephanidis (Ed.). *HCI International 2011 Posters’ Extended Abstracts* (pp. 284-288). Berlin, Germany: Springer.

Küller, R., Mikellides. B., & Janssens. J. (2009). Color, arousal, and performance - a comparison of three experiments. *Color Research & Application, 34*, 141-52. <https://doi.org/10.1002/col.20476>

Kumi, R., Conway, C. M., Limayem, M., & Goyal, S. (2013). Learning in color: how color and affect influence learning outcomes. *IEEE Transactions on Professional Communications, 56*, 2-15. <https://doi.org/10.1109/TPC.2012.2208390>

Lichtenfeld, S., Elliot, A. J., Maier, M. A, & Pekrun, R. (2012). Fertile green: green facilitates creative performance. *Personality & Social Psychology Bulletin, 38*, 784-97. <https://doi.org/10.1177/0146167212436611>

Lichtenfeld, S., Maier, M. A., Elliot, A. J., & Pekrun, R. (2009). The semantic red effect: Processing the word red undermines intellectual performance. *Journal of Experimental Social Psychology, 45*, 1273-1276. <https://doi.org/10.1016/j.jesp.2009.06.003>

Mehta, R., & Zhu, R. J. (2009). Blue or red? Exploring the effect of color on cognitive task performances. *Science, 323*, 1226-1229. <https://doi.org/10.1126/science.1169144>

Meyer, M. J., & Bagwell, J. (2012). The non-impact of paper color on exam performance. *Issues in Accounting Education*, *27*, 691-706. <https://doi.org/10.2308/iace-50142>

Rutchick, A. M., Slepian, M. L., & Ferris, B. D. (2010). The pen is mightier than the word: object priming of evaluative standards. *European Journal of Social Psychology, 40*, 704-708. <https://doi.org/10.1002/ejsp.753>

Schmidt, D. R., Ruskell, T. G., & Kohl, P. B. (2013). Effect of paper color on students’ physics exam performances. *AIP Conference Proceedings, 1513*. <https://doi.org/10.1063/1.4789730>

Steele, K. M. (2014). Failure to replicate the Mehta and Zhu (2009) color-priming effect on anagram solution times. *Psychonomic Bulletin & Review, 21*, 771-776. <https://doi.org/10.3758/s13423-013-0548-3>

Tal, I. R., Akers, K. G., & Hodge, G. K. (2008). Effect of paper color and question order on exam performance. *Teaching of Psychology, 35*, 26-28. <https://doi.org/10.1080/00986280701818482>

Thorstensen, C. A. (2012). *Effects of color perception and enacted avoidance behavior on intellectual task performance in an achievement context* (Unpublished master’s thesis). Appalachian State University, Boone, NC.

Xia, T., Song, L., Wang, T. T., Tan, L., & Mo, L. (2016). Exploring the effect of red and blue on cognitive task performances. *Frontiers in Psychology, 7* (784). <https://doi.org/10.3389/fpsyg.2016.00784>

Yamazaki, A. K. (2010). An analysis of background-color effects on the scores of a computer-based English test. In R. Setchi, I. Jordanov, R. J. Howlett, & L. C. Jain (Eds.), *Proceedings of the Knowledge-Based and Intelligent Information and Engineering Systems 14^th^ International Conference KES 2010*. (pp. 630-636). Berlin, Germany: Springer. <https://doi.org/10.1007/978-3-642-15390-7_65>

Yamazaki, A. K., & Eto, K. (2011). A preliminary examination of background-color effects on the scores of computer-based English grammar tests using near-infrared spectroscopy. In A. König, A. Dengel, K. Hinkelmann, K. Kise, R. J. Howlett, & L. C. Jain (Eds.), *Proceedings of the Knowledge-Based and Intelligent Information and Engineering Systems 15^th^ International Conference KES 2011*. (pp. 630-636). Berlin, Germany: Springer. <https://doi.org/10.1007/978-3-642-23854-3_4>

# References for Included Studies

Arthur, W., Cho, I., & Muñoz, G. J. (2016). Red vs. green: Does the exam booklet color matter in higher education summative evaluations? Not likely*. Psychonomic Bulletin & Review, 23*, 1596-1601. <https://doi.org/10.3758/s13423-016-1009-6>

Bertrams, A., Baumeister, R. F., Englert, C., & Furley, P. (2015). Ego depletion in color priming research: self-control strength moderates the detrimental effect of red on cognitive test performance. *Personality and Social Psychology Bulletin*, *41*, 311-322. <https://doi.org/10.1177/0146167214564968>

Caschera, D. (2015) The impact of colour perception on cognitive task performance. *The Huron University College Journal of Learning and Motivation, 53*(1). Retrieved from <http://ir.lib.uwo.ca/hucjlm/vol53/iss1/2>

von Castell, C., Stelzmann, D., Oberfeld, D., Welsch, R., & Hecht, H. (2018). Cognitive performance and emotion are indifferent to ambient color. *Color Research & Application, 43*, 65-74. <https://doi.org/10.1002/col.22168>

Drummond, R. R. (2017). Color perception: Assessing the effects of red on cognitive-task performance. *Scholars: Journal of Undergraduate Research, 29*. Retrieved from <http://www.mckendree.edu/academics/scholars/issue-29.php>

Elliot, A. J., Maier, M. A., Moller, A. C., Friedman, R., & Meinhardt, J. (2007). Color and psychological functioning: The effect of red on performance attainment*. Journal of Experimental Psychology: General, 136*, 154-168. <https://doi.org/10.1037/0096-3445.136.1.154>

Elliot, A. J., & Thorstenson, C. A. (2019). The influence of perceiving the colors red and green on analytical performance: A within-participants test. *North American Journal of Psychology, 21*, 517-524.

Gnambs, T., Appel, M., & Batinic, B. (2010). Color red in web-based knowledge testing. *Computers in Human Behavior, 26*, 1625-1631. <https://doi.org/10.1016/j.chb.2010.06.010>

Gnambs, T., Appel, M., & Kaspar, K. (2015). The effect of the color red on encoding and retrieval of declarative knowledge. *Learning and Individual Differences, 42*, 90-96. <https://doi.org/10.1016/j.lindif.2015.07.017>

Hulshof, B. (2013). *The influence of colour and scent on people’s mood and cognitive performance in meeting rooms* (Unpublished master’s thesis). University of Twente, Netherlands. Retrieved from <http://purl.utwente.nl/essays/63446>

Larsson, E. E., & von Stumm, S. (2015). Seeing red? The effect of colour on intelligence test performance. *Intelligence, 48*, 133-136. <https://doi.org/10.1016/j.intell.2014.11.007>

Maier, M. A., Elliot, A. J., & Lichtenfeld, S. (2008). Mediation of the negative effect of red on intellectual performance. *Personality and Social Psychology Bulletin, 34*, 1530-1540. <https://doi.org/10.1177/0146167208323104>

Pedley, A. (2016). *The influence of red stimuli on cognitive performance in achievement context settings* (Unpublished doctoral dissertation). Guildford, England: University of Surrey. Retrieved from <http://epubs.surrey.ac.uk/id/eprint/811661>

Shi, J., Zhang, C., & Jiang, F. (2015). Does red undermine individuals' intellectual performance? A test in China. *International Journal of Psychology, 50*, 81-84. <https://doi.org/10.1002/ijop.12076>

Smajic, A., Merritt, S., Banister, C., & Blinebry, A. (2014). The red effect, anxiety, and exam performance: a multistudy examination. *Teaching of Psychology, 41*, 37-43. <https://doi.org/10.1177/0098628313514176>

Steele, K. M., Intriago, P., Stenborg, A., Roper, M., Pennington, J., Vettorazi. S., & Kirschner, B. (2015, November). *Does the color red reduce performance in an achievement task?* Poster presented at the 56th meeting of the Psychonomic Society, Chicago, IL.

Steele, K. M., Fisher, J., Novachek, A., Retarides, J., Vernon, A., Orr, W., & Martin, Z. (2016, November). *The effect of red on performance in an achievement task: A second attempt*. Poster presented at the 57th meeting of the Psychonomic Society, Boston, MA.

Steele, K. M., Sablan, N., Roper, M., Mitchell, J., Novachek, A., Retarides, J., & Stowe, A. (2017, November). *The effect of red on performance in an achievement task: Third failure to replicate*. Poster presented at the 58th meeting of the Psychonomic Society, Vancouver, BC.

Steele, K. M., Amador, M. N., Easter, L., & Ross, A. I. (2018, November). *The effect of red on performance in an achievement task: Fourth failure to replicate*. Poster presented at the 59th meeting of the Psychonomic Society, New Orleans, LA.

Thorstenson, C. A. (2015). Functional equivalence of the color red and enacted avoidance behavior? *Social Psychology, 46*, 306-311. <https://doi.org/10.1027/1864-9335/a000245>

Vuković, Z. K., & Petrović, I. B. (2017). The effect of red color on anagram performance - replication of experiment. In O. Tosković, K. Damnjanović, & L. Lazarević (Eds.), *Proceedings of the XIII Scientific Conference Studies in Psychology* (pp. 10-14). Belgrade, Serbia: University of Belgrade.

Zhang, T., & Han, B. (2014). Experience reverses the red effect among Chinese stockbrokers. *PloS One, 9*(2). <https://doi.org/10.1371/journal.pone.0089193>

# Coding Process of Primary Studies

In a standardized coding protocol all relevant variables including their range of potential values were defined. Two coders independently extracted the relevant information from the identified publications. The focal information was the effect of the experimental manipulation on cognitive test performance, that is, the standardized mean difference between red color and a control color. In addition, the size of the examined sample was recorded. Moreover, various information on potential moderators of the color effect was coded. This included, among others, the type of the administered cognitive measure (anagrams, reasoning, knowledge), the study design (between- or within-subject), and the control color (green, blue, gray, other). Moreover, it was recorded whether the two colors were matched on hue and lightness^[[1]](#footnote-2)^, whether the color manipulation was presented on paper or a computer screen, and whether the color manipulation was only presented before the test or also during testing. The entire coding protocol is available below (Table A2). The coding process was evaluated with two-way intraclass coefficients (ICC; Shrout & Fleiss, 1979) that indicate strong intercoder reliability for values exceeding .70 and excellent reliability for values greater than .90 (LeBreton & Senter, 2008). The intercoder reliability for the focal statistics and the sample size fell between ICC = .96, 95% CI[.95, .97] and ICC = 1.00, 95% CI [1.00, 1.00], respectively. The author resolved disagreements by revisiting the respective study.

References

LeBreton, J. M., & Senter, J. L. (2008). Answers to 20 questions about interrater reliability and interrater agreement. *Organizational Research Methods, 11*, 815-852. <https://doi.org/10.1177/1094428106296642>

Shrout, P. E., & Fleiss, J. L. (1979). Intraclass correlations: Uses in assessing rater reliability. *Psychological Bulletin, 86*, 420-428. <https://doi.org/10.1037/0033-2909.86.2.420>

Table A2.

*Coding Guide.*

| **Variable** | **Description** | **Value** | **Example** |
| --- | --- | --- | --- |
| study | Study ID: last name of first author + publication year (in case of multiple samples within one study: append a single letter) | open text | schmidt2012 |
| pubyear | Publication year | value range: [1900, 2017] | 2012 |
| sno | Unique ID for each sample | value range: [1,[ | 1 |
| mno | Unique number of measure within sample | value range: [1,[ | 1 |
| cntry | Country of origin of participants as ISO code | open text | DE |
| pubtype | Publication type | 1= peer-reviewed journal, 2= presentation, thesis | 1 |
| manip | Description of the experimental manipulation | open text | Cover sheet |
| color | Control color | open text | green |
| n1 | Sample size for red color condition | value range: [1,[ | 15 |
| n2 | Sample size for control color condition | value range: [1,[ | 15 |
| n | Total sample size if sample sizes for each color condition are not reported | value range: [1,[ | 30 |
| sample | Description of sample | open text | Undergraduates |
| female | Percentage of women in sample (%) | value range: [0,100] | 50 |
| age | Mean age (in years) of participants | value range: [5,[ | 20 |
| m1 | Mean of red color condition | value range: ],[ | 10 |
| sd1 | Standard deviation of red color condition | value range: ],[ | 2 |
| m2 | Mean of control color condition | value range: ],[ | 10 |
| sd2 | Standard deviation of control color condition | value range: ],[ | 2 |
| t | t-statistic for test of experimental manipulation | value range: ],[ | 3 |
| F | F-statistic for test of experimental manipulation (with df = 1) | value range: ],[ | 3 |
| d | Cohen's d, only if means, standard deviations, or t-values are not reported | value range: [0,[ | 0,5 |
| stat | Any other statistical result for the calculation of an effect size | open text | F(1, 10) = 0.50 |
| page | Page of publication reporting the statistics | open text | p11 |
| rec | Invert outcome because lower values indicate better performance | 0 = no 1 = yes | 0 |
| test | Description of cognitive measure | open text | Anagrams |
| testcat | Cognitive measure categorized | 1 = Reasoning 2 = Anagrams 3 = Knowledge |  |
| matched | Were the colors matched on hue and lightness? | 0 = no 1 = yes | 1 |
| mode | Presentation mode of color manipulation | 0 = paper-based 1 = computer-based 2 = other | 0 |
| dur | Duration of color presentation | 0 = only before test 1 = also during test | 0 |
| design | Study design | 0 = between-subject 1 = within-subject | 0 |
| setting | Test setting | 0 = individual 1 = group | 0 |
| note | General comments | open text |  |

1. It was also coded whether the matching procedure for the two colors was checked with a spectrophotometer (Elliot & Maier, 2014). Because only a single study was identified that used this method, this moderator was not further examined. [↑](#footnote-ref-2)
