# Supplementary material for: Limited evidence for the effect of red color on cognitive performance: A meta-analysis
Source: Psychon Bull Rev. 2020 Jul 7;27(6):1374–82. doi: 10.3758/s13423-020-01772-1 (PMC7704521; doi:10.3758/s13423-020-01772-1)
Supplement: Supplementary file 2 — (DOCX 94.7 kb) [file 13423_2020_1772_MOESM2_ESM.docx]

Supplement B: Characteristics of Samples Included in the Meta-Analysis

The coded data is also available in the data repository at <https://osf.io/nhmsc/>.

| Study | Country | Publ. | Design | Manip. | Color | *N* | %♀ | Age | Measure | Matched | Mode | Duration | *d* | *V_d_* |
| --- | --- | --- | --- | --- | --- | --- | --- | --- | --- | --- | --- | --- | --- | --- |
| Arthur et al. (2016)  *Study 1* | US | Yes | B | Color of cover sheet | Green | 76 |  |  | Knowledge | No | Paper | Before | 0.43 | 0.05 |
| Arthur et al. (2016)  *Study 2* | US | Yes | B | Color of all sheets | Green | 164 |  |  | Knowledge | No | Paper | During | 0.02 | 0.02 |
| Arthur et al. (2016)  *Study 3* | US | Yes | B | Color of all sheets | Green | 87 |  |  | Knowledge | No | Paper | During | 0.08 | 0.05 |
| Bertrams et al. (2015)  *Experiment 1* | DE | Yes | B | Colored rectangle on cover sheet | Gray | 33 | 58 | 22 | Reasoning | No | Paper | Before | 0.04 | 0.12 |
| Bertrams et al. (2015)  *Experiment 2* | DE | Yes | B | Colored rectangle on cover sheet | Gray | 38 | 79 | 22 | Reasoning | No | Paper | Before | 0.36 | 0.11 |
| Caschera (2015)  Easy test | US | No | B | Ink color of entire test | Green | 16 | 53 | 27 | Anagrams | No | Paper | During | -0.41 | 0.26 |
| Caschera (2015)  Difficult test | US | No | B | Ink color of entire test | Green | 16 | 53 | 27 | Anagrams | No | Paper | During | -1.06 | 0.29 |
| von Castell et al. (2018) | DE | Yes | B | Color of room | White | 87 | 51 | 19 | Reasoning | No | Paper | During | -0.16 | 0.05 |
|  |  |  |  |  | Blue | 82 |  |  | Reasoning |  |  |  | 0.06 | 0.05 |
|  |  |  |  |  | White | 87 |  |  | Reasoning |  |  |  | 0.63 | 0.05 |
|  |  |  |  |  | Blue | 82 |  |  | Reasoning |  |  |  | 0.24 | 0.05 |
| Drummond (2017) | US | No | B | Ink color of items | Green | 61 | 73 | 20 | Anagrams | No | Paper | During | -0.54 | 0.07 |
|  |  |  |  |  | Black | 80 |  |  | Anagrams |  |  |  | -0.09 | 0.05 |
| Elliot et al. (2007)  *Experiment 1* | US | Yes | B | Ink color of respondent ID on each page | Green | 46 | 75 | 20 | Anagrams | No | Paper | During | -0.08 | 0.09 |
|  |  |  |  |  | Black | 44 |  |  | Anagrams |  |  |  | -0.20 | 0.09 |
| Elliot et al. (2007)  *Experiment 2* | DE | Yes | B | Colored rectangle on cover sheet | Green | 30 | 91 | 22 | Reasoning | No | Paper | Before | -1.24 | 0.16 |
|  |  |  |  |  | White | 31 |  |  | Reasoning |  |  |  | -1.00 | 0.15 |
| Elliot et al. (2007)  *Experiment 3* | DE | Yes | B | Colored rectangle on cover sheet | Green | 22 | 83 | 17 | Reasoning | No | Paper | Before | -1.51 | 0.23 |
|  |  |  |  |  | Gray | 18 |  |  | Reasoning |  |  |  | -0.76 | 0.24 |
| Elliot et al. (2007)  *Experiment 4* | DE | Yes | B | Colored rectangle on cover sheet | Green | 40 | 56 | 18 | Reasoning | Yes | Paper | Before | -0.53 | 0.10 |
|  |  |  |  |  | Gray | 35 |  |  | Reasoning |  |  |  | -0.48 | 0.12 |
| Elliot & Thorstenson (2019)  *Experiment 1* | US | Yes | W | Test color of items | Green | 40 | 82 | 20 | Anagram | No | Computer | During | 0.10 | 0.10 |
| Elliot & Thorstenson (2019)  *Experiment 2* | US | Yes | W | Test color of items | Green | 135 | 69 | 25 | Anagram | No | Computer | During | 0.11 | 0.03 |
| Gnambs et al. (2010)  *Study 1* | AT | Yes | B | Progress bar of web survey | Green | 131 | 69 | 25 | Knowledge | Yes | Computer | During | -0.51 | 0.03 |
| Gnambs et al. (2010)  *Study 2* | DE | Yes | B | Submit button of web survey | Blue | 133 | 52 | 24 | Knowledge | Yes | Computer | During | -0.04 | 0.03 |
|  |  |  |  |  | Blue | 128 |  |  | Knowledge |  |  |  | -0.08 | 0.03 |
| Gnambs et al. (2015) | AT | Yes | B | Colored rectangle on cover sheet | Gray | 83 | 66 | 17 | Knowledge | Yes | Paper | Before | -0.09 | 0.04 |
| Hulshof (2013) | NL | No | B | Color of room walls | Blue | 122 | 40 | 30 | Reasoning | No | Paper | During | -0.36 | 0.03 |
| Larsson & von Stumm (2015) | UK | Yes | B | Ink color of respondent ID on each page | Green | 185 | 52 | 35 | Reasoning | No | Paper | During | -0.12 | 0.02 |
|  |  |  |  |  | Green | 187 |  |  | Knowledge |  |  |  | -0.10 | 0.02 |
|  |  |  |  |  | Green | 187 |  |  | Reasoning |  |  |  | -0.04 | 0.02 |
|  |  |  |  |  | Green | 187 |  |  | Reasoning |  |  |  | 0.03 | 0.02 |
|  |  |  |  |  | Green | 187 |  |  | Knowledge |  |  |  | 0.09 | 0.02 |
| Maier et al. (2008):  *Experiment 1* | DE | Yes | B | Colored rectangle on cover sheet | Gray | 20 | 45 |  | Reasoning | Yes | Paper | Before | -1.37 | 0.25 |
| Maier et al. (2008):  *Experiment 3* | DE | Yes | B | Colored rectangle on cover sheet | Gray | 22 | 41 |  | Reasoning | Yes | Paper | Before | -0.96 | 0.20 |
| Pedley (2016)  *Experiment 2* | UK | No | B | Colored rectangle before test | Blue | 42 | 57 | 33 | Reasoning | Yes | Computer | Before | 0.02 | 0.10 |
|  |  |  |  |  | Green | 42 |  |  | Reasoning |  |  |  | 0.16 | 0.10 |
|  |  |  |  |  | Gray | 42 |  |  | Reasoning |  |  |  | -0.36 | 0.10 |
| Pedley (2016)  *Experiment 3* | UK | No | B | Background color of computer screen | Blue | 88 | 46 | 34 | Reasoning | Yes | Computer | During | 0.05 | 0.05 |
|  |  |  |  |  | Green | 88 |  |  | Reasoning |  |  |  | 0.16 | 0.05 |
| Pedley (2016)  *Experiment 4* | UK | No | B | Colored rectangle on cover sheet | Blue | 30 | 78 | 17 | Reasoning | Yes | Paper | Before | 0.61 | 0.14 |
|  |  |  |  |  | Green | 30 |  |  | Reasoning |  |  |  | 0.28 | 0.13 |
|  |  |  |  |  | Gray | 30 |  |  | Reasoning |  |  |  | 0.39 | 0.14 |
| Pedley (2016)  *Experiment 5* | UK | No | B | Colored rectangle on cover sheet | White | 32 | 100 | 20 | Reasoning | Yes | Paper | Before | -1.22 | 0.15 |
| Pedley (2016)  *Experiment 6* | UK | No | B | Colored rectangle on cover sheet | Green | 30 | 81 | 19 | Reasoning | Yes | Paper | Before | 0.22 | 0.13 |
|  |  |  |  |  | White | 30 |  |  | Reasoning |  |  |  | 0.49 | 0.14 |
| Shi et al. (2014) | CN | Yes | B | Color of items | Blue | 58 | 86 | 20 | Reasoning | No | Computer | During | -0.57 | 0.07 |
| Smajic et al. (2013)  *Study 1* | US | Yes | B | Color of cover sheet | Green | 137 |  |  | Knowledge | No | Paper | Before | -0.16 | 0.03 |
| Smajic et al. (2013)  *Study 2: Class 1* | US | Yes | B | Color of cover sheet and color mark on each page | Blue | 31 |  |  | Knowledge | No | Paper | During | 0.02 | 0.13 |
|  |  |  |  |  | Green | 32 |  |  | Knowledge |  |  |  | -0.02 | 0.13 |
|  |  |  |  |  | White | 30 |  |  | Knowledge |  |  |  | 0.09 | 0.14 |
| Smajic et al. (2013)  *Study 2: Class 2* | US | Yes | B | Color of cover sheet and color mark on each page | Blue | 25 |  |  | Knowledge | No | Paper | During | 0.45 | 0.17 |
|  |  |  |  |  | Green | 24 |  |  | Knowledge |  |  |  | 0.11 | 0.17 |
|  |  |  |  |  | White | 20 |  |  | Knowledge |  |  |  | 0.22 | 0.24 |
| Steele et al. (2015) | US | No | B | Colored rectangle before test | Gray | 206 | 64 |  | Anagrams | Yes | Computer | Before | -0.12 | 0.02 |
|  |  |  |  |  | Green | 203 |  |  | Anagrams |  |  |  | 0.07 | 0.02 |
| Steele et al. (2016) | US | No | B | Colored rectangle before test | Gray | 163 | 74 |  | Anagrams | Yes | Computer | Before | -0.06 | 0.02 |
|  |  |  |  |  | Green | 188 |  |  | Anagrams |  |  |  | -0.04 | 0.02 |
| Steele et al. (2017) | US | No | B | Colored rectangle before test | Gray | 266 | 71 |  | Anagrams | Yes | Computer | Before | -0.07 | 0.02 |
|  |  |  |  |  | Green | 246 |  |  | Anagrams |  |  |  | -0.01 | 0.02 |
| Steele et al. (2018) | US | No | B | Colored rectangle before test | Gray | 282 | 79 |  | Anagrams | Yes | Computer | Before | -0.01 | 0.01 |
|  |  |  |  |  | Green | 277 |  |  | Anagrams |  |  |  | -0.08 | 0.01 |
| Thorstenson (2015) | US | Yes | B | Text color of instruction | Green | 82 | 68 |  | Anagrams | Yes | Paper | Before | -0.47 | 0.05 |
|  |  |  |  |  | Gray | 86 |  |  | Anagrams |  |  |  | -0.37 | 0.05 |
| Vuković & Petrović (2017) | RS | No | B | Ink color of respondent ID on each page | Black | 96 | 87 | 22 | Anagrams | No | Paper | During | 0.07 | 0.04 |
|  |  |  |  |  | Green | 95 |  |  | Anagrams |  |  |  | 0.17 | 0.04 |
| Zhang & Han (2014) | CN | Yes | B | Colored rectangle before test | Green | 24 | 0 | 23 | Reasoning | Yes | Computer | Before | -1.11 | 0.19 |
| *Note*. Publ. = Published in an academic journal (*yes*) or as a conference presentation or thesis (*no*). Design = Between- (B) or within-subject (W) design. Manip. = Implementation of the experimental manipulation. Color = Control color. %♀ = Percentage of female respondents. Age = Mean age of sample (in years). Measure = Type of cognitive performance test. Matched = Colors were matched on hue and lightness (*yes*) or not (*no*). Mode = Presentation mode of experimental manipulation. Duration = Colors were only presented before the test (*before*) or also during the test (*during*). *d* = Effect size as standardized mean difference (negative effects indicate worse performance in the red condition; see Supplement C). *V_d_* = Sampling variance of *d*. | | | | | | | | | | | | | | |

# Homogeneity of Variances

The homogeneity of variances between the color conditions was examined for the 52 comparisons that reported means and standard deviations in each condition. The *F*-tests with adjusted *p*-values (Holm, 1979) for the respective variance ratios identified significantly (*p* < .05) smaller variances in the red condition for two effects (2.9% of all effects) reported in Drummond (2017) and Thorstenson (2015). Because no systematic different variances were observed for most studies, the mean differences were standardized with regard to their pooled variance (see Supplement C) as compared to the variance in the control condition (i.e., Glass Δ; Hedges & Olkin, 1985).

References

Hedges, L. V., & Olkin, I. (1985). *Statistical Methods for Meta-Analysis*. Orlando, FL: Academic Press.

Holm, S. (1979). A simple sequentially rejective multiple test procedure. *Scandinavian Journal of Statistics, 6*, 65-70.
