# Supplementary material for: Limited evidence for the effect of red color on cognitive performance: A meta-analysis
Source: Psychon Bull Rev. 2020 Jul 7;27(6):1374–82. doi: 10.3758/s13423-020-01772-1 (PMC7704521; doi:10.3758/s13423-020-01772-1)
Supplement: Supplementary file 3 — (DOCX 97.1 kb) [file 13423_2020_1772_MOESM3_ESM.docx]

Supplement C: Effect Size Calculation

Content

Calculation of Effect Sizes 2

Calculation of Sampling (Co)Variances for Effect Sizes 4

Calculation of Averaged Effect Sizes Within Samples 5

References 6

# Calculation of Effect Sizes

The meta-analysis pooled standardized mean differences between two color conditions. The respective effect sizes were calculated following Hedges (1981) as

$d=\frac{M_{red}-M_{control}}{SD_{pooled}}$ (B1)

with *M_red_* as the mean test score in the red color condition and *M_control_* as the mean score in the control color condition. If a study used multiple control colors, an effect size was calculated for each control color. The pooled standard deviation *SD_pooled_* for between-subject designs was derived as

$SD_{pooled}=\sqrt{\frac{\left(n_{red}-1\right)\cdot Var_{red}+\left(n_{control}-1\right)\cdot Var_{control}}{n_{red}+n_{control}-2}}$ (B2)

with *Var_red_* and *Var_control_* as the variances in the red color and control color conditions and *n_red_* and *n_control_* indicating the respective sample sizes. For studies that reported only the total sample size but not the sample sizes of each color condition, equal samples were assumed in both groups. For within-subject designs *SD_pooled_* was calculated as

$SD_{pooled}=\sqrt{\frac{Var_{red}+Var_{control}}{2}}$, (B3)

which has been argued to lead to effect sizes that are more comparable between different study designs and, thus, should be preferred for meta-analyses involving within- and between-subject experiment as compared to other standardization schemes (Lakens, 2013).

If no means and variances in the different color conditions were reported, *d* was calculated from the *t*-statistic (Rosnow & Rosenthal, 2003) as

$d=t\cdot\sqrt{\frac{n_{red}+n_{control}}{n_{red}\cdot n_{control}}}$. (B4)

Similar, effect sizes were derived from *F*-statistics with 1 degree of freedom using the transformation *t* = -*F*^0.5^ and applying (B4).

Following Hedges and Olkin (1985), a large-sample approximation of *d* was derived as *d^*^* = *d* ∙*J* with

$J=1-\frac{3}{4\cdot\left(n_{red}+n_{control}\right)-9}$. (B5)

# Calculation of Sampling (Co)Variances for Effect Sizes

The sampling variance of *d^*^* was approximated by (Hedges & Olkin, 1985)

. (B6)

Because some studies implemented multiple control colors (e.g., green and gray), covariances between the two dependent effect sizes $d_{1}^{*}$ and $d_{2}^{*}$ within a study (i.e., for different control colors) was derived following Gleser and Olkin (2009) as

$Cov(d_{1}^{*},d_{2}^{*})=\frac{1}{n_{red}}+\frac{d_{1}^{*}\cdot d_{2}^{*}}{2\cdot n_{total}}$ (B7)

where $n_{total}=n_{red}+n_{control_{1}}+n_{control_{2}}$ is the total sample size. One study (Larsson & von Stumm, 2015) administered multiple outcomes using a single control color. The covariances between these effect sizes $d_{1}^{*}$ and $d_{2}^{*}$ were calculated (Gleser & Olkin, 2009) as

$Cov(d_{1}^{*},d_{2}^{*})=\left(\frac{1}{n_{red}}+\frac{1}{n_{control}}\right)\cdot r+\frac{d_{1}^{*}\cdot d_{2}^{*}}{2\cdot\left(n_{red}+n_{control}\right)}\cdot r^{2}$ (B8)

where *r* is the sample correlation between the two outcomes. Because *r* was not reported a value of .5 was imputed that reflected typical correlations between cognitive measures in previous research (e.g., Singer & Strasser, 2017).

# Calculation of Averaged Effect Sizes Within Samples

For analyses based on within-sample averaged effect sizes, a weighted mean effect size was calculated for each sample. Let **Y** be the *K* x 1 vector of *K* dependent effect sizes within a given sample and **V** the *K* x *K* sampling variance-covariance matrix for these effect sizes that were calculated as described above. The inverse-variance weighted mean effect size for this sample is given as

$\bar{\mathbf{Y}}=\bar{\mathbf{V}}\mathbf{1}'\mathbf{V}^{-1}\mathbf{Y}$ (B9)

with **1** being a *K* x 1 vector of 1s. Then, the sampling variance of $\bar{\mathbf{V}}$ is

$\bar{\mathbf{V}}=1/\left(\mathbf{1}'\mathbf{V}^{-1}\mathbf{1}\right)$. (B10)

Note, if the sampling variances *Var*(*d*^*^) for all *k* effect sizes are equal and a constant sampling correlation ρ between all effect sizes is assumed B9 and B10 can be simplified to

$\begin{matrix} \bar{\mathbf{Y}}=\frac{1}{K}\sum_{j=1}^{K} Y_{j} \\ \bar{\mathbf{V}}=\frac{\left(K-1\right)\rho+1}{K}\cdot Var\left(d^{*}\right) \end{matrix}$ (B11)

(Borenstein, Hedges, Higgins, & Rothstein, 2009). Because no such assumptions were made, the more general forms presented in B9 and B10 were used in the present meta-analysis. A more detailed description of the formal model is given in Pustejovsky (2019).

# References

Borenstein, M., Hedges, L. V., Higgins, J. P. T., Rothstein, H. R. (2009). *Introduction to Meta-Analysis*. Chichester, UK: John Wiley. <https://doi.org/10.1002/9780470743386>

Gleser, L. J., & Olkin, I. (2009). Stochastically dependent effect sizes. In H. Cooper, L. V. Hedges, & J. C. Valentine (Eds.), *The handbook of research synthesis and meta-analysis* (2nd ed., pp. 357–376). New York, NY: Russell Sage Foundation.

Hedges, L. V. (1981). Distribution theory for Glass's estimator of effect size and related estimators. *Journal of Educational Statistics, 6*, 107-128. <https://doi.org/10.3102/10769986006002107>

Hedges, L. V., & Olkin, I. (1985). *Statistical methods for meta-analysis*. New York, NY: Academic Press.

Lakens, D. (2013). Calculating and reporting effect sizes to facilitate cumulative science: a practical primer for t-tests and ANOVAs. *Frontiers in Psychology, 4* (863). <https://dx.doi.org/10.3389/fpsyg.2013.00863>

Larsson, E. E., & von Stumm, S. (2015). Seeing red? The effect of colour on intelligence test performance. *Intelligence, 48*, 133-136. <https://doi.org/10.1016/j.intell.2014.11.007>

Pustejovsky, J. E. (2019). *Sometimes, aggregating effect sizes is fine*. Retrieved from [https://web.archive.org/web/20190723001908/https://www.jepusto.com/sometimes-aggregating-effect-sizes-is-fine/](https://web.archive.org/web/20190723001908/https:/www.jepusto.com/sometimes-aggregating-effect-sizes-is-fine/)

Rosnow, R. L., & Rosenthal, R. (2003). Effect sizes for experimenting psychologists. *Canadian Journal of Experimental Psychology, 57*, 221-237. <https://doi.org/10.1037/h0087427>

Singer, V., & Strasser, K. (2017). The association between arithmetic and reading performance in school: A meta-analytic study. *School Psychology Quarterly, 32*, 435-448. <http://dx.doi.org/10.1037/spq0000197>
