# Supplementary material for: Limited evidence for the effect of red color on cognitive performance: A meta-analysis
Source: Psychon Bull Rev. 2020 Jul 7;27(6):1374–82. doi: 10.3758/s13423-020-01772-1 (PMC7704521; doi:10.3758/s13423-020-01772-1)
Supplement: Supplementary file 4 — (DOCX 67.8 kb) [file 13423_2020_1772_MOESM4_ESM.docx]

Supplement D: Meta-Analytic Results

Content

Meta-Analytic Results and Subgroup Analyses 2

Sensitivity Analyses 3

Additional Moderator Analyses 5

# Meta-Analytic Results and Subgroup Analyses

Table D1.

*Meta-Analysis of the Effect of Red Color on Cognitive Performance.*

|  |  |  |  | Observed effect | | Pooled effect | | |  |  |
| --- | --- | --- | --- | --- | --- | --- | --- | --- | --- | --- |
|  | *k*_1_ | *k*_2_ | *$\tilde{N}$* | *d* | *SD_d_* | Δ | *SE*_Δ_ | 95% CI_Δ_ | τ | *I*^2^ |
| Overall | 67 | 38 | 76 | -0.16 | 0.47 | -0.13^*^ | 0.05 | [-0.23, -0.03] | 0.21^*^ | 0.49 |
| *Cognitive measure* (*Q*_m_ = 2.22, *df* = 2, *p* = .329; *Q*_e_ = 110.13, *df* = 64, *p* < .001) | | | | | | | | | | |
| Anagrams ^a^ | 20 | 12 | 96 | -0.15 | 0.29 | -0.06 | 0.05 | [-0.15, 0.03] | - | - |
| Reasoning | 31 | 17 | 38 | -0.26 | 0.62 | -0.34^*^ | 0.14 | [-0.61, -0.06] | 0.48^*^ | 0.77 |
| Knowledge | 16 | 10 | 92 | 0.03 | 0.23 | -0.04 | 0.07 | [-0.18, 0.10] | 0.12 | 0.24 |
| *Control color* (*Q*_m_ = 1.05, *df* = 3, *p* = .789; *Q*_e_ = 111.89, *df* = 63, *p* < .001) | | | | | | | | | | |
| Green | 32 | 28 | 84 | -0.19 | 0.42 | -0.10 | 0.06 | [-0.22, 0.01] | 0.19^*^ | 0.46 |
| Blue | 11 | 9 | 82 | 0.04 | 0.33 | -0.03 | 0.10 | [-0.23, 0.16] | 0.17 | 0.35 |
| Gray ^a^ | 14 | 14 | 40 | -0.28 | 0.49 | -0.12^*^ | 0.05 | [-0.22, -0.01] | - | - |
| Other | 10 | 9 | 38 | -0.12 | 0.59 | -0.13 | 0.17 | [-0.46, 0.20] | 0.40^*^ | 0.67 |
| *Method effects* (*Q*_m_ = 2.05, *df* = 3, *p* = .561; *Q*_e_ = 109.19, *df* = 63, *p* < .001) | | | | | | | | | | |
| Colors matched | 30 | 17 | 84 | -0.18 | 0.48 | -0.20^*^ | 0.08 | [-0.36, -0.04] | 0.25^*^ | 0.62 |
| Colors not matched | 37 | 21 | 61 | -0.14 | 0.47 | -0.08 | 0.07 | [-0.21, 0.06] | 0.20^*^ | 0.42 |
| Paper-based | 47 | 26 | 40 | -0.18 | 0.53 | -0.17^*^ | 0.08 | [-0.34, -0.01] | 0.33^*^ | 0.64 |
| Computer-based ^a^ | 20 | 12 | 132 | -0.12 | 0.31 | -0.08 | 0.05 | [-0.17, 0.01] | - | - |
| Manipulation before test | 34 | 21 | 41 | -0.26 | 0.57 | -0.23^*^ | 0.10 | [-0.43, -0.03] | 0.38^*^ | 0.75 |
| Manipulation during test | 33 | 18 | 87 | -0.05 | 0.31 | -0.07 | 0.06 | [-0.18, 0.04] | 0.12 | 0.25 |
| *Note*. *k*_1_ = Number of effects sizes; *k*_2_ = Number of samples; *$\tilde{N}$* = Median sample size per effect; *d* = Mean unweighted Cohen’s *d*; *SD_d_* = Standard deviation of *d*; Δ = Pooled inverse variance weighted Cohen’s *d*; *SE*_Δ_ = Standard error of Δ; 95% CI_Δ_ = 95% confidence interval of Δ; τ = Random effect (standard deviation); *I*^2^ = Proportion of random variance in Δ due to between-sample heterogeneity; *Q*_m_ = Omnibus test of moderators (Hedges & Pigott, 2004); *Q*_e_ = Test for residual heterogeneity.  Negative values of *d* and Δ indicated lower scores for red color as compared to the control color.  ^a^ Fixed-effects model.  ^*^ *p* < .05 | | | | | | | | | | |

# Sensitivity Analyses

Because outlier analyses using the studentized residuals and Cook’s distance (Viechtbauer & Cheung, 2010) did not identify influential cases, it is unlikely that outliers substantially affected the results. However, sensitivity analyses evaluated to what degree selected effects affected the overall pooled effect. First, the effect sizes were averaged within sample (see Supplement C) to derive independent effect sizes. As summarized in Table D2, a univariate meta-analysis on these averaged effects yields exactly the same results as the multivariate meta-analysis summarized in Table D1. Thus, both approaches are equivalent. Then, the meta-analyses were also repeated excluding samples with within-subject designs (i.e., Elliot & Thorstenson, 2019) or studies reporting multiple outcomes (i.e., Larsson & von Stumm, 2015). However, these analyses yielded rather similar results as compared to the complete case analyses. Therefore, it is unlikely that these effects substantially distorted the overall pooled effect. Finally, samples conducted in high-stakes settings (e.g., real exams) were excluded, that is samples 1 and 2 in Arthur et al. (2016) and the samples reported in Smajic et al. (2014). These analyses showed a slightly larger effect (Δ = -0.17) as compared to the full case analyses (Δ = -0.13). Unfortunately, this setting effect is confounded with the administered cognitive measure (i.e., knowledge tests). Therefore, the reason for the slightly different results remains unclear.

References

Viechtbauer, W., & Cheung, M. W.-L. (2010). Outlier and influence diagnostics for meta-analysis. *Research Synthesis Methods, 1*, 112-125. <https://doi.org/10.1002/jrsm.11>

Table D2.

*Sensitivity Analyses for Within-Sample Averaged Effect Sizes.*

|  |  |  | Observed effect | | Pooled effect | | |  |
| --- | --- | --- | --- | --- | --- | --- | --- | --- |
|  | *k* | *$\tilde{N}$* | *d* | *SD_d_* | Δ | *SE*_Δ_ | 95% CI_Δ_ | τ |
| Overall | 38 | 80 | -0.24 | 0.50 | -0.13^*^ | 0.05 | [-0.23, -0.03] | 0.21^*^ |
| Excluding within-subject designs | 36 | 80 | -0.26 | 0.51 | -0.15^*^ | 0.06 | [-0.26, -0.04] | 0.23^*^ |
| Excluding multiple outcomes studies | 37 | 76 | -0.24 | 0.50 | -0.14^*^ | 0.06 | [-0.25, -0.03] | 0.23^*^ |
| Excluding high-stake settings | 33 | 84 | -0.29 | 0.51 | -0.17^*^ | 0.06 | [-0.29, 0.06] | 0.24^*^ |
| *Note*. *k* = Number of within-sample averaged effects sizes; *$\tilde{N}$* = Median sample size per sample; *d* = Mean unweighted Cohen’s *d*; *SD_d_* = Standard deviation of *d*; Δ = Pooled inverse variance weighted Cohen’s *d*; *SE*_Δ_ = Standard error of Δ; 95% CI_Δ_ = 95% confidence interval of Δ; τ = Random effect (standard deviation).  Negative values of *d* and Δ indicated lower scores for red color as compared to the control color.  ^*^ *p* < .05 | | | | | | | | |

# Additional Moderator Analyses

Some authors (Gnambs et al., 2010; Ioan et al., 2007) demonstrated gender-specific differences in the red color effect. Following stereotype threat theory and evolutionary theory, they argued that men should be more susceptible to color effects than women. To evaluate this proposition the percentage of female respondents was used as a moderator in the present meta-analysis. For the 32 studies reporting the respective information no gender effect was observed, *Q_m_*(*df* = 1) = 0.51, *p* = .477. This does not provide evidence for previously identified gender-specific red color effects.

References

Gnambs, T., Appel, M., & Batinic, B. (2010). Color red in web-based knowledge testing. *Computers in Human Behavior, 26*, 1625-1631. <https://doi.org/10.1016/j.chb.2010.06.010>

Ioan, S., Sandualche, M., Avramescu, S., Ilie, A., Neacsu, A., Zagrean, L., & Moldovan, M. (2007). Red is a distractor for men in competition*. Evolution and Human Behavior, 28*, 285-293. <https://doi.org/10.1016/j.evolhumbehav.2007.03.001>
