# Supplementary material for: Limited evidence for the effect of red color on cognitive performance: A meta-analysis
Source: Psychon Bull Rev. 2020 Jul 7;27(6):1374–82. doi: 10.3758/s13423-020-01772-1 (PMC7704521; doi:10.3758/s13423-020-01772-1)
Supplement: Supplementary file 5 — (DOCX 194 kb) [file 13423_2020_1772_MOESM5_ESM.docx]

Supplement E: Analyses of Publication Bias

A major threat to valid inference from meta-analyses is a failure to include unpublished studies with small effects because statistically significant findings are more likely to being submitted and accepted for publication (Rothstein, Sutton, & Borenstein, 2005). The presence and consequence of a potential publication bias was examined in several ways including (a) a visual inspection of the funnel plots, (b) testing the funnel plot for asymmetry, (c) estimating meta-analytic selection models, and (d) examining the *p*-value distribution of the effect sizes.

**Visual inspection of funnel plots**. The funnel plot visualizes the observed effect sizes and their standard error (Sterne & Egger, 2001). In the absence of publication bias the individual effect sizes are expected to be distributed around the pooled effect and form the shape of an inverted funnel; that is, studies with larger samples (and, thus, smaller standard errors) should closely cluster around the pooled effect near to the top of the figure, whereas small-sample studies (with larger standard errors) are expected to be more strongly distributed around the pooled effect near the bottom of the plot. In the presence of publication bias small studies with less precision are expected to by systematically missing and create an asymmetrical shape of the funnel plot; thus, no (or rather few) effects are displayed in the bottom right-hand side of the plot. To improve their interpretability, funnel plots can be enhanced by contours of statistical significance; thus, a 95% or 99% pseudo confidence interval region is drawn around the null effect. These contour-enhanced funnel plots for anagram, reasoning, and knowledge tests are displayed in Figure 3 of the main paper. Both plots indicated highly asymmetric shapes. A similar picture also emerged when evaluating all effect sizes for the three cognitive measure together (see Figure E1). Small studies that did not support an effect of red color on cognitive performance were missing, whereas studies with larger samples were more evenly distributed.


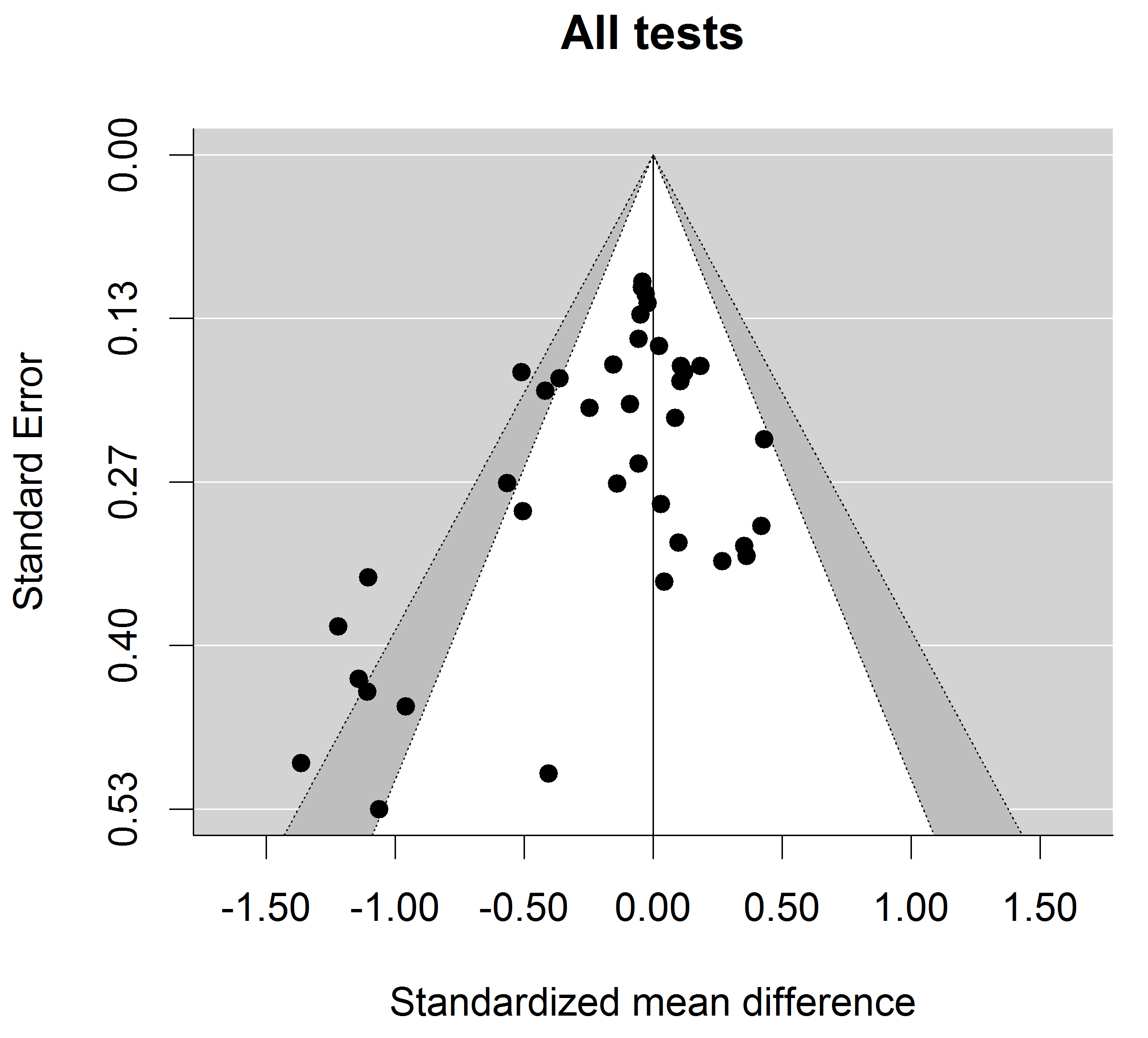


*Figure E1*. Funnel plots with 95% (white) and 99% (gray) confidence intervals for all effect sizes across the three cognitive measures.

**Tests for funnel plot asymmetry**. Following Begg and Mazumdar (1994) the funnel plot of the effects sizes in the meta-analytic database was tested for asymmetry by calculating the rank correlation between the effect sizes and their standard errors (see Table 2). A significant negative correlation would indicate systematically missing studies that might have distorted the pooled effect. The respective correlations indicated a significant (*p* < .05) publication bias for reasoning tests (*r* = -.43, *p* = .017). For the other cognitive measures, no significant effects were identified. Regression tests following Peters and colleagues (Peters, Sutton, Jones, Abrams, & Rushton, 2006) corroborated this result. Regressing the observed effect sizes on the inverse of their sample sizes estimated significant effects (see Table 2), thus, also indicating asymmetric funnel plots. The intercepts in these regressions represent the pooled effect for an infinitesimal sample size and, thus, an estimate of the pooled effect corrected for publication bias. The corrected pooled effects were close to 0 for anagram test and even slightly positive for reasoning tests (indicating an effect in the opposite direction). Surprisingly, for knowledge tests slightly larger (albeit not significant) effects were estimated. Because the performance of regression tests for the identification of publication bias is still in dispute, two additional variants were used. First, following Stanley (2017) the effect sizes were either regressed on the sampling variances (PEESE) or the respective standard deviations (PET). If the corrected effect in the PET model was significant at a 10% level, these results were evaluated and the PEESE results otherwise. Second, because the sampling variance of Cohen’s *d* depends on the value of *d* (see Supplement C) modified PET / PEESE analyses were also conducted that substituted the sampling variance with a value of 4 / *n* (see Pustejovsky & Rodner, 2019). These results (see Table 2) yielded rather similar results. Albeit for the knowledge test that exhibited no publication bias, theses analyses seemed to overcorrect the pooled effects and suggested *better* performance in the red color condition. In summary, for no cognitive measure a significant effect of red color was estimated. This suggests that a publication bias might have overestimated the reported findings in the main paper. However, it should be noted that other explanations for funnel plot asymmetry are also possible (see Lau, Ioannidis, Terrin, Schmid, & Olkin, 2006).

**Selection models**. The weight-function model by Vevea and Woods (2005) was used to explore the effect of different selection mechanisms on the estimated pooled effects. For reasoning tests these models indicated substantial (*p* < .05) selection effects (see Table 2). More importantly, they identified no effect of red color on anagram test performance, Δ = 0.08. Again, these analyses were repeated substituting the sampling variance of *d* with a value of 4 / *n* (see Pustejovsky & Rodner, 2019). However, this resulted in nearly identical results (see Table 2). These results indicate that the color effect for reasoning tests reported in the main paper might represent an overestimation of the true effect.

**Distribution of *p*-values**. The evidential value for a reported effect can be scrutinized by evaluating the *p*-values associated with the effect sizes. This approach uses the idea that the distribution of *p*-values conditional on the population effect size should be uniform to determine whether the published findings provide evidence for a true phenomenon or, rather, are no more than a reflection of publication bias (van Assen, van Aeart, & Wicherts, 2015; Simonsohn, Nelson, & Simmons, 2014; Simonsohn, Simmons, & Nelson, 2015). Unfortunately, contemporary methods such as *p-curve* (Simonsohn et al., 2014) or *puniform* (van Assen et al., 2015) have some drawbacks such as their use of only statistically significant effects or the ignorance of between-study heterogeneity (McShane et al., 2016; van Aert, Wicherts, & van Assen, 2016). Therefore, a new method called *puniform** (van Aert & van Assen, 2020) was used which is a refinement of an approach initially introduced by van Assen and colleagues (2015) to estimate a pooled effect corrected for publication bias. An advantage of *puniform** is that it is also applicable to random-effects models and is not limited to statistically significant results. For anagram and knowledge tests, these analyses did not indicate publication bias (*L_pb_* = 0.13, *p* = .939, and *L_pb_* = 0.86, *p* = .649). However, the adjusted effect sizes from these analyses indicated no substantial color effect (Δ = -0.03, *p* = .494, and Δ = -0.01, *p* = .850). In contrast, for the reasoning test *puniform** identified significant publication bias (*L_pb_* = 6.78, *p* = .034). Again, the estimated effect size adjusted for publication bias indicated no substantial effect of red color, Δ = 0.03, *p* = .704.

Taken together, the different analyses reported here suggest that publication bias seemed to have distorted the publicly available research findings on red color effects and reasoning. After taking into account publication bias, most analyses evidenced no effect of red color at all.

References

Begg, C. B., & Mazumdar, M. (1994). Operating characteristics of a rank correlation test for publication bias. *Biometrics, 50*, 1088-1101. <https://doi.org/10.2307/2533446>

Coburn, K. M., & Vevea, J. L. (2017). *weightr: EstimatingWeight-Function Models for Publication Bias* (R package version 1.1.2). URL: <https://CRAN.R-project.org/package=weightr>

Lau, J., Ioannidis, J. P. A., Terrin, N., Schmid, C. H., & Olkin, I. (2006). Evidence based medicine: The case of the misleading funnel plot. *BMJ: British Medical Journal, 333*, 597-600. <https://doi.org/10.1136/bmj.333.7568.597>

McShane, B. B., Böckenholt, U., & Hansen, K. T. (2016). Adjusting for publication bias in meta-analysis: An evaluation of selection methods and some cautionary notes. *Perspectives on Psychological Science, 11*, 730-749. <https://doi.org/10.1177/1745691616662243>

Peters, J. L., Sutton, A. J., Jones, D. R., Abrams, K. R., & Rushton, L. (2006). Comparison of two methods to detect publication bias in meta-analysis. *Jama, 295*, 676-680. <https://doi.org/10.1001/jama.295.6.676>

Rothstein, H. R., Sutton, A. J., & Borenstein, M. (2005). Publication bias in meta-analysis. In H. R. Rothstein, A. J. Sutton, & M. Borenstein (Eds.), *Publication bias in meta- analysis: Prevention, assessment, and adjustments* (pp. 1-8). West Sussex, England: John Wiley & Sons.

Simonsohn, U., Simmons, J. P., & Nelson, L. D. (2015). Better p-curves: Making p-curve analysis more robust to errors, fraud, and ambitious p-hacking, a reply to Ulrich and Miller (2015). *Journal of Experimental Psychology: General, 144*, 1146-1152. <https://doi.org/10.1037/xge0000104>

Simonsohn, U., Nelson, L. D., & Simmons, J. P. (2014). P-curve: A key to the file-drawer. *Journal of Experimental Psychology. General, 143*, 534-547. <https://doi.org/10.1037/a0033242>

Sterne, J. A. C., & Egger, M. (2001). Funnel plots for detecting bias in meta-analysis: Guidelines on choice of axis. *Journal of Clinical Epidemiology, 54*, 1046–1055. <https://doi.org/10.1016/S0895-4356(01)00377-8>

Van Aert, R. C. M., & van Assen, M. A. L. M. (2020). Correcting for publication bias in a meta-analysis with the p-uniform* method. *BITSS Preprints*. <https://doi.org/10.31222/osf.io/zqjr9>

Van Aert, R. C. M., Wicherts, J. M., & van Assen, M. A. L. M. (2016). Conducting meta-analyses on p-values: Reservations and recommendations for applying p-uniform and p-curve. *Perspectives on Psychological Science, 11*, 713-729. <https://doi.org/10.1177/1745691616650874>

Van Assen, M. A. L. M., van Aert, R. C. M., & Wicherts, J. M. (2015). Meta-analysis using effect size distributions of only statistically significant studies. *Psychological Methods, 20*, 293-309. <https://doi.org/10.1037/met0000025>

Vevea, J. L., & Woods, C. M. (2005). Publication bias in research synthesis: Sensitivity analysis using a priori weight functions. *Psychological Methods, 10*, 428-443. <https://doi.org/10.1037/1082-989X.10.4.428>
